# Effects of the Adsorbed Dispersant Layer on Steric Stabilization in Multicomponent Nickel Pastes

**DOI:** 10.3390/molecules31162885

**Published:** 2026-08-18

**Authors:** Seong-Yeon Park, Ju Young Kim, Gayoung Yoo, Seon-Hee Park, Taesung Kim, Kang-Sahn Kim, Shin’ichi Higai, Gi Joo Bang, Hong-Seok Kim

**Affiliations:** 1 Advanced Component Development Lab, Corporate R&D Institute, Samsung Electro-Mechanics Co., Ltd., Suwon 16674, Gyeonggi-do, Republic of Korea; seongyeon.park@samsung.com (S.-Y.P.); jyday.kim@samsung.com (J.Y.K.); gayoung.yoo@samsung.com (G.Y.); kangsahn.kim@samsung.com (K.-S.K.); giwoong.ha@samsung.com (S.H.); 2 MLCC Process Architecture Lab, Component Business Unit, Samsung Electro-Mechanics Co., Ltd., Suwon 16674, Gyeonggi-do, Republic of Korea; seonhee0426.park@samsung.com (S.-H.P.); tscv.kim@samsung.com (T.K.)

**Keywords:** dispersant, nickel paste, steric stabilization, crystallinity, molecular dynamics, cryogenic focused ion beam-scanning electron microscopy, potential of mean force, multilayer ceramic capacitors

## Abstract

In multilayer ceramic capacitors, the internal electrodes are fabricated by printing thin layers of nickel paste, which contains a dispersant to prevent the agglomeration of nickel powder particles within the paste. We used molecular dynamics simulations and cryogenic focused ion beam-scanning electron microscopy to investigate the effects of microscopic structures in the adsorbed dispersant layer on the steric stabilization of nickel powder particles. Three dispersants were considered with different molecular structures. The simulation results indicated that stearic acid (SA) resulted in the highest steric stabilization efficiency, followed by lauric acid (LA) and oleic acid (OA). The high crystallinity of the SA and LA layers resulted in compressed molecular chains that induced strong repulsion between nickel powder particles, and the high effective thickness of the SA layer induced a stronger repulsion. The OA layer offered less steric stabilization because the molecular chains exhibited interpenetration rather than compression. The experimental results confirmed that the steric stabilization of nickel paste samples qualitatively aligned with the simulation results. Thus, multicomponent nickel pastes containing a polar solvent require a dispersant that forms a thick and highly crystalline adsorbed layer on nickel powder particles for effective steric stabilization.

## 1. Introduction

Multilayer ceramic capacitors (MLCCs) are a key passive component in electronic applications ranging from mobile devices and automotive electronics to high-performance computing [1,2], which has driven demand for their miniaturization while maintaining a high capacitance and long-term reliability [3,4]. To satisfy these requirements, the internal electrodes of MLCCs are fabricated by printing thin layers of nickel paste several hundred nanometers thick. The nickel paste comprises nickel powder along with binders, solvents, and dispersants [5,6,7,8]. The nickel powder within the paste must be dispersed uniformly to ensure continuity of the electrodes. Dispersants adsorb onto the surface of nickel powder via anchoring groups, while their tail groups induce the steric stabilization required to prevent agglomeration [9,10,11,12,13,14]. These dispersants are completely removed in the subsequent debinding process through thermal decomposition and volatilization, along with binders and solvents [15]. Previous studies on selecting dispersants generally tested simplified nickel powder suspensions without binders, which provided insights into the effects of interaction affinity between dispersants and solvents on steric stabilization, and various dispersants such as polyacrylamide, oligomeric polyesters, polyvinylpyrrolidone, fatty acids, and glycerol trioleate have been reported to be effective [16,17,18,19,20,21]. However, a nickel paste is a complex system where individual constituents engage in multiscale interactions. Thus, dispersants are selected through case studies that explore various combinations of binders, solvents, and dispersants that may not fully account for the actual steric stabilization observed in a nickel paste [5,6]. The decoupling of these complex interactions into specific mechanisms is essential for establishing a physics-informed design strategy to enhance steric stabilization in a nickel paste.

From a molecular design perspective, the performance of a dispersant is governed by its molecular structure, which typically comprises an anchoring group and a tail group. Research has characterized the effects of anchoring groups, including functional groups [22], acidity/basicity [23], and ionic characteristics [24]. However, the influence of tail groups remains relatively underexplored [18]. Although the affinity of anchoring groups can be quantified experimentally by spectroscopic analyses [22,23,24], the microscopic behavior of tail groups remains inherently difficult to probe directly in a nickel paste, which makes their effect on steric stabilization less clear.

In this study, we used molecular dynamics (MD) to simulate the effects of the dispersant tail groups on the steric stabilization of a nickel paste. To achieve this goal, we constructed a multicomponent model comprising a nickel powder, polymeric binder, solvent, and dispersant. The microscopic structures of the adsorbed dispersant layer were analyzed to investigate the characteristics linked to steric stabilization efficiency. Potential of mean force (PMF) calculations were performed to quantify interactions between two nickel powder particles to elucidate the influence of structures in the adsorbed dispersant layer on steric stabilization at the atomistic level [25,26]. To enable simulations that are both computationally feasible and comparable to experiments, the model assumes that all dispersant molecules strongly chemisorb onto the nickel surface [17,27,28,29], with the adsorption density adjusted to compensate for the size difference between simulated and experimental particles. In experiments, nickel pastes formulated with the same constituents as in the simulations were evaluated for the degree of nickel powder agglomeration. While rheological properties are commonly used to evaluate paste dispersibility, the rheological behavior of multicomponent pastes is also influenced by the interactions and dynamics of binder polymers and solvent molecules [30,31]. Therefore, it can be difficult to isolate the specific effects of dispersant molecular structure solely from macroscopic rheological measurements. Accordingly, this study focuses on the steric stabilization mechanisms induced by the adsorbed dispersant layer. The resulting dispersion state was qualitatively assessed through cryogenic focused ion beam-scanning electron microscopy (Cryo FIB-SEM) analysis. By comparing the qualitative trends of our simulation and experimental results, we elucidated the effects of microscopic structures in the adsorbed dispersant layer on the steric stabilization of the nickel paste, offering essential insights into the underlying mechanisms.

## 2. Results and Discussion

### 2.1. Characterization of the Adsorbed Dispersant Layer

**Figure 1:**
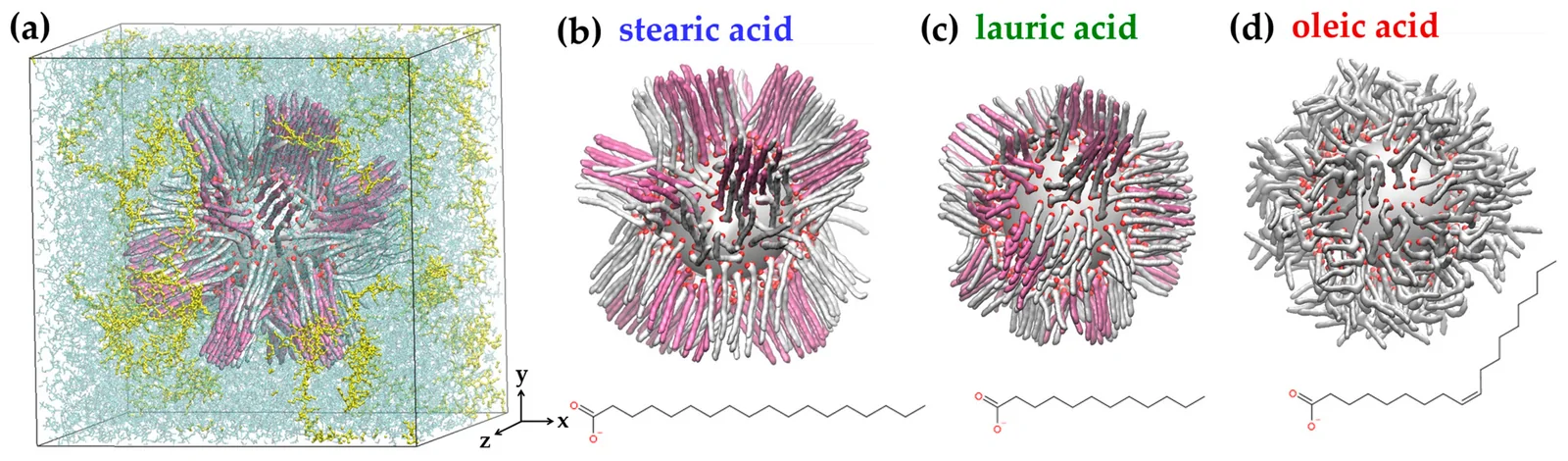
MD simulation for the structural characterization of the adsorbed dispersant layer: (**a**) Representative snapshot of a dispersant adsorbed on a nickel powder particle in a solvent (dihydroterpinyl acetate, DHTA) containing binder molecules (ethyl cellulose, EC). Color coding: dispersant tail groups (crystallized: pink, not crystallized: white), dispersant anchoring groups (red), polymeric binder (yellow), and solvent (light blue). Detailed organization of adsorbed dispersant layers: (**b**) stearic acid, (**c**) lauric acid, and (**d**) oleic acid. The binder and solvent molecules are omitted for clarity. The chemical structures of each dispersant are provided at the bottom of each panel.

We analyzed the microscopic structures of the adsorbed dispersant layer on the nickel powder surface to investigate which characteristics can influence steric stabilization. Figure 1a shows the constructed model, which comprises a dispersant layer on a nickel powder particle in a solvent–binder environment. Different dispersants were selected with different molecular structural characteristics, including the carbon chain length and bond type: stearic acid (SA, C18:0), lauric acid (LA, C12:0), and oleic acid (OA, C18:1). The notation Cn:m denotes the number of carbon atoms (n) and double bonds (m). Figure 1b–d show that even minor variations in the molecular structure of the dispersant led to distinct configurations of the adsorbed dispersant layer. The SA layer exhibited a semicrystalline structure characterized by partially ordered packing of linear SA molecules. The LA layer had a similar structure but with reduced thickness, consistent with the shorter carbon chain length. In contrast, the OA layer exhibited a highly disordered structure, attributed to molecular bending induced by its cis double bond.

**Figure 2:**
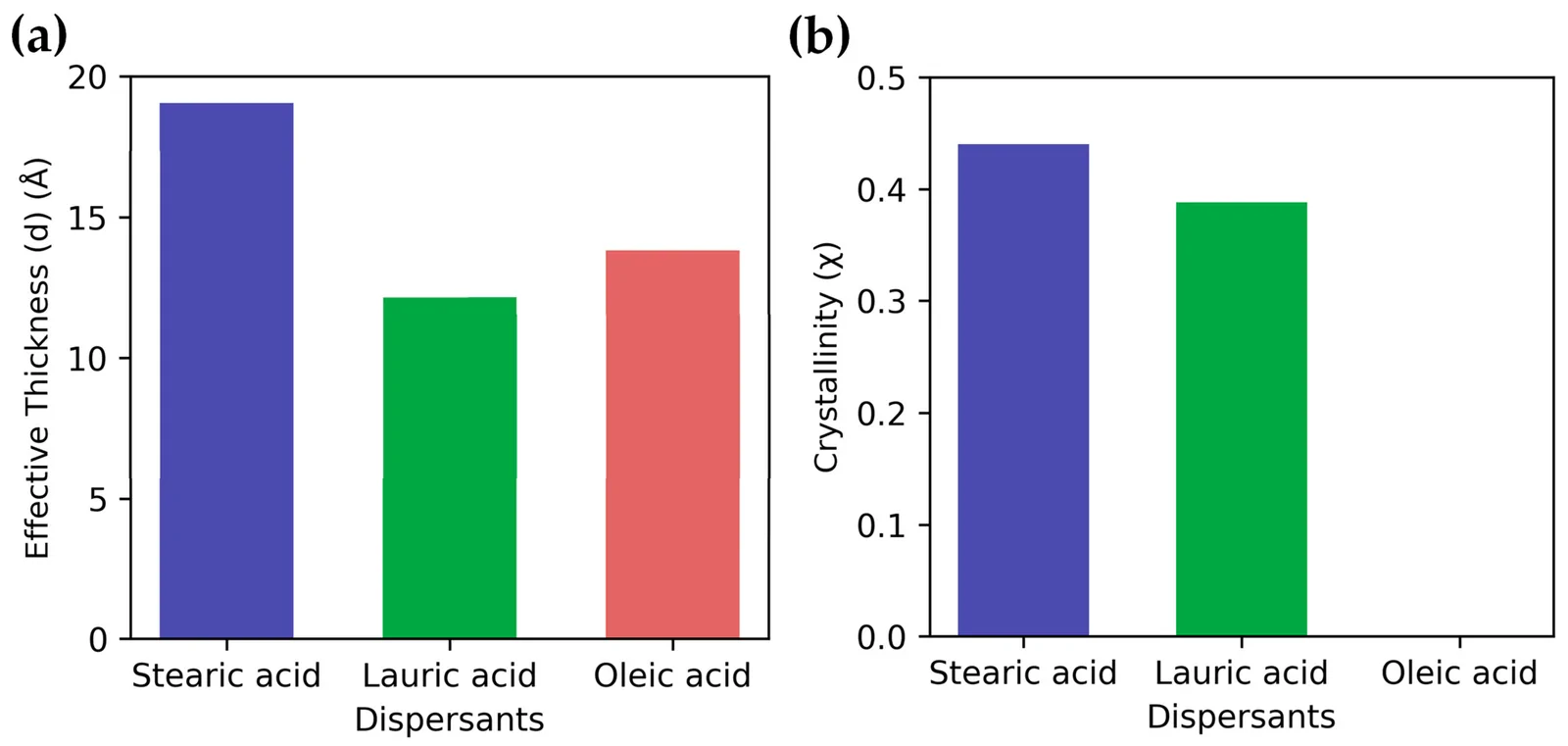
Quantitative characterization of the adsorbed dispersant layers: (**a**) Effective thickness, which was defined as the median distance of the terminal atom of the dispersant molecule from the nickel powder surface. (**b**) Crystallinity, which was defined as the proportion of dispersant molecules in the semicrystalline packing state within the adsorbed dispersant layer.

Following the morphological analysis of the adsorbed dispersant layers, we focused on two key features known to influence steric stabilization: the effective thickness and crystallinity. A sufficiently thick dispersant layer can prevent nickel powder particles from agglomerating by effectively counteracting van der Waals attractive forces [32,33]. Meanwhile, a low crystallinity of the dispersant layer has been suggested to be effective for steric stabilization because crystallized dispersant molecules can induce attractive interactions between nickel powder particles [34]. Thus, we quantified the effective thickness and crystallinity of the adsorbed dispersant layers to predict the steric stabilization state of the nickel paste. As shown in Figure 2a, the effective thicknesses of the SA, LA, and OA layers were calculated as 19.0, 12.1, and 13.8 Å, respectively. The thicker SA layer should be more effective for steric stabilization than the LA and OA layers. As shown in Figure 2b, the crystallinities of the SA, LA, and OA layers were calculated as 0.44, 0.39, and 0, respectively. These results indicate that the OA layer should be more effective for steric stabilization owing to its zero crystallinity, while the SA layer should have the least effectiveness. Based on these conflicting trends, the effective thickness and crystallinity are likely insufficient to explain the effect of microscopic structures in the adsorbed dispersant on steric stabilization in the nickel paste. Consequently, direct quantification of the steric stabilization was required to elucidate its relationship with these two features.

### 2.2. Effects of Microscopic Structures on Steric Stabilization Efficiency

**Figure 3:**
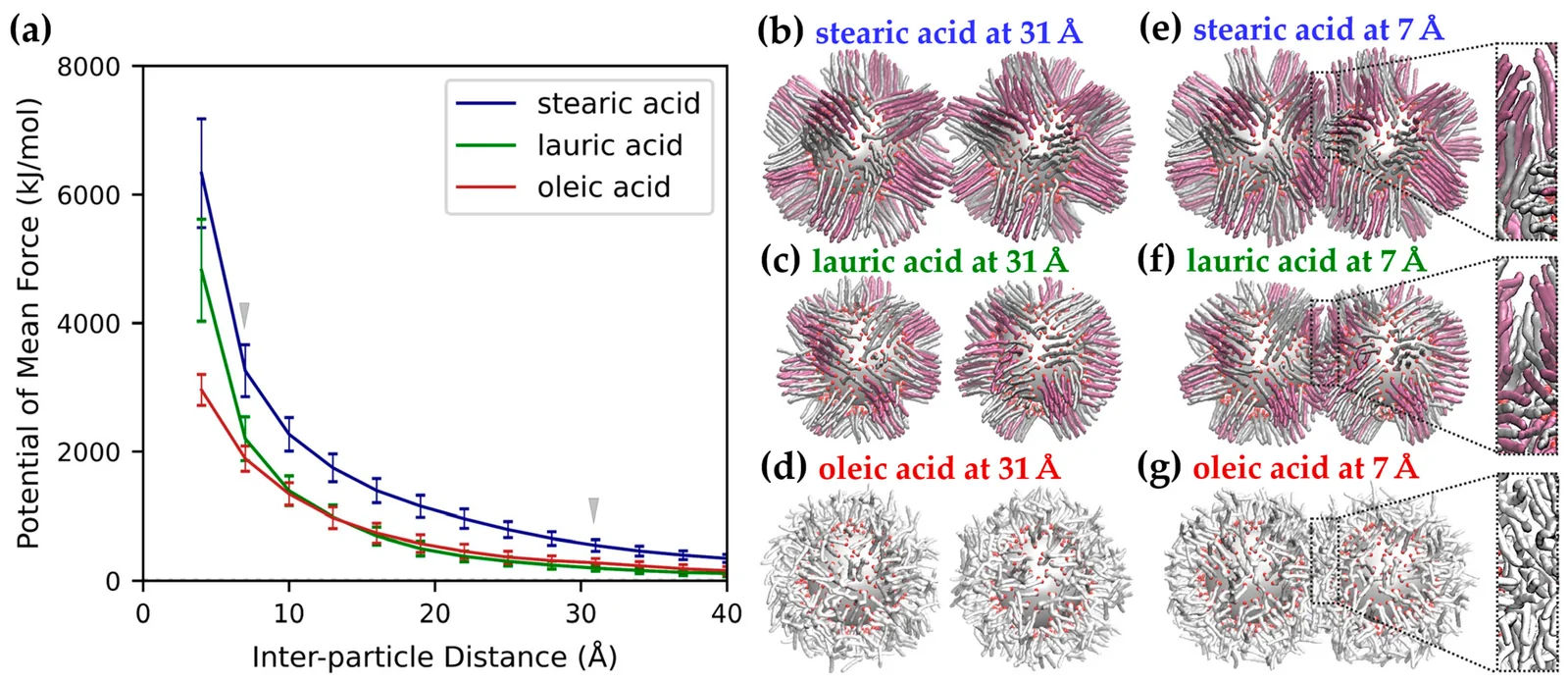
MD simulation results: (**a**) Calculated PMF as a function of the interparticle separation distance for SA (blue), LA (green), and OA (red). Error bars represent the standard error obtained from 12 independent simulations. Snapshots of MD simulations illustrating the morphology of the adsorbed dispersant layers on two nickel powder particles at a large separation distance (31 Å) and a small separation distance (7 Å): (**b**,**e**) SA, (**c**,**f**) LA, and (**d**,**g**) OA. The insets on the right side of (**e**–**g**) show magnified views of the adsorbed dispersant layer at a separation distance of 7 Å. Binder and solvent molecules were omitted for clarity.

The steric stabilization efficiency of the adsorbed dispersant layers was evaluated by quantifying the repulsive energy barrier between powder particles using PMF as a function of the separation distance. Based on colloidal physics, stronger steric stabilization at the particle surface increases the reversible work required for particles to approach each other, which results in a higher PMF [25,26]. As shown in Figure 3a, the SA layer exhibited the highest PMF at all distances, which indicates the most effective steric stabilization. While the LA and OA layers exhibit comparable PMFs at large separation distances (≥10 Å), the former displayed a higher PMF at closer separation distances (<10 Å). This suggests that the LA layer exhibits more effective steric stabilization than the OA layer despite its lower effective thickness and higher crystallinity, which have been considered unfavorable for steric stabilization in previous studies [32,33]. To further understand these results in terms of established descriptors for steric stabilization at the atomic scale, the molecular behavior of the adsorbed dispersant layers was analyzed. As shown in Figure 3b–d, only the SA layer exhibited chain overlap at a separation distance of 31 Å, which suggests that the high effective thickness of the SA layer induced chain overlap even at large separation distances and resulted in strong repulsion. However, because the LA and OA layers did not exhibit chain overlap at large separation distances, further investigation at closer separation distances was required to understand the effects of their microscopic structures on steric stabilization. As shown in Figure 3e–g, all three dispersant layers exhibited chain overlap at a separation distance of 7 Å. Whereas the chains of the OA layer exhibited interpenetration, the chains of the SA and LA layers were compressed and vertically oriented. These compressed structures likely generate strong repulsion upon steric hindrance, which resulted in higher PMFs than for the OA layer. The SA layer exhibited a higher PMF than the LA layer, which can be attributed to its higher effective thickness [32,33]. The chains in the OA layer can mix freely to avoid severe compression, which minimizes structural resistance and explains why it achieved the lowest PMF. Consequently, the crystalline structures of the SA and LA layers provide stronger steric stabilization owing to their compressed chains, and the greater effective thickness of SA further amplified repulsion.

Based on these results, effective steric stabilization for a nickel paste was concluded to require a dispersant that forms a thick and highly crystalline adsorbed layer on nickel powder particles. While the influence of the effective thickness is consistent with previous studies [32,33], the influence of crystallinity contradicts a previous study that reported high crystallinity reduces steric stabilization [34]. This difference can be attributed to the effects of solvent polarity on the miscibility of the dispersant. While the nonpolar solvent used in the previous study (i.e., hexane) prefers saturated chains, the polar solvent used here (i.e., DHTA) is more compatible with unsaturated chains.

### 2.3. Experimental Validation

**Figure 4:**
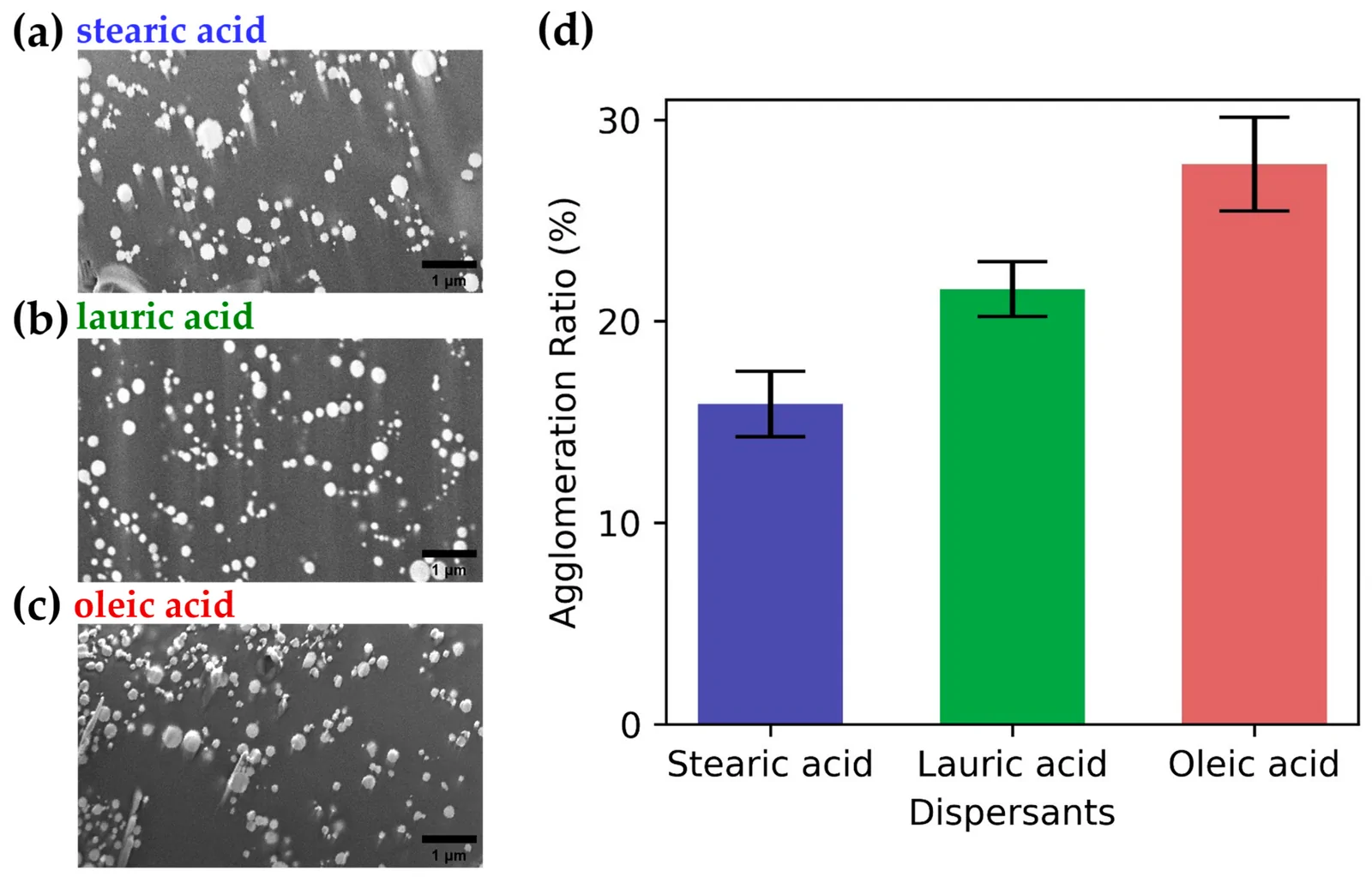
Experimental observation of nickel powder agglomeration in pastes containing different dispersants: Representative Cryo FIB-SEM images of preserved nickel pastes in the liquid state with different dispersants: (**a**) SA, (**b**) LA, and (**c**) OA. The white particles correspond to nickel powder, and the background gray area corresponds to organic materials comprising the solvent and binder. (**d**) Agglomeration ratio (%) for each dispersant, which was defined as the number of agglomerated particles relative to the total number of observed nickel powder particles. Nickel powder particles were considered agglomerated if the center-to-center distance was within the median particle diameter (*D*_50_ = 137.1 nm). A lower agglomeration ratio was associated with enhanced steric stabilization.

For experimental validation of simulation results, we analyzed the dispersion state of nickel powder in paste. However, the dispersion state may change as the paste samples dry. Therefore, we used Cryo FIB-SEM to observe the cross-sections of samples preserved in a liquid state [35,36,37], as shown in Figure 4a–c. To quantitatively evaluate steric stabilization, nickel powder particles with a center-to-center distance of ≤D_50_ (~137 nm) were defined as agglomerated. The agglomeration ratio was calculated as the number of agglomerated particles divided by the total number of particles. As shown in Figure 4d, the agglomeration ratios with SA, LA, and OA were 0.16, 0.22, and 0.28, respectively. This indicates that the steric stabilization efficiency of nickel paste was highest with SA, followed by LA and OA, which is qualitatively consistent with the simulation.

## 3. Materials and Methods

### 3.1. Computational Methodology

#### 3.1.1. Simulation Framework

All MD simulations were carried out using the Large-scale Atomic/Molecular Massively Parallel Simulator (LAMMPS) [38,39]. To describe atomic interactions, the Dreiding force field [40] was employed in its united-atom form, which facilitated the computationally efficient simulations required for equilibration. Under this force field, the total potential energy was modeled as the sum of bonded (i.e., bond, angle, dihedral, and improper) and nonbonded (i.e., van der Waals and hydrogen bond) interactions. Specifically, van der Waals interactions were truncated at a cutoff distance of 10.0 Å by using Lorentz–Berthelot mixing rules [41] with all other parameters adopted from the original Dreiding definition [40]. To construct the model, a nickel powder particle was simplified as a virtual sphere with no interactions and a diameter of 5 nm to improve computational efficiency. This assumption of no interaction is justified because fatty acids are known to adsorb strongly onto nickel/nickel oxide surfaces [17,27,28,29]. Therefore, the interactions between nickel and other constituents are considered to be effectively screened by the adsorbed dispersant layer. Meanwhile, although the experimental nickel powder has a median diameter D50 of 137 nm, atomistic simulation at this scale is computationally unfeasible, leading to the modeling of a 5 nm virtual sphere. However, the high curvature of the 5 nm sphere is expected to result in a lower tail-end density relative to the adsorption density than that expected for the 137 nm powder [42]. To compensate for this curvature difference, the number of adsorbed dispersant molecules on the 5 nm sphere was adjusted so that the resulting tail-end density matched the theoretical target density estimated for the 137 nm particle size. This adjustment resulted in a higher adsorption density on the 5 nm sphere than that corresponding to the 137 nm particle size. The anchoring groups of the dispersant were equidistantly distributed on the surface of this virtual sphere by using the Fibonacci Sphere algorithm [43]. Positional constraints were applied to these anchoring sites, but the virtual sphere was permitted to rotate about its center. In contrast, the dispersant tail groups, solvent molecules, and binder molecules were treated as fully flexible to ensure realistic thermodynamic behavior. Thermodynamic equilibrium was maintained in the NVT ensemble at 300 K by using a Nosé–Hoover thermostat [44,45] with a time step of 1.0 fs. Periodic boundary conditions were applied in all three Cartesian directions to simulate a continuous bulk environment. The simulation box size was set to be sufficiently large to avoid periodic boundary artifacts. To verify that the model had reached an equilibrium state, potential energy convergence was confirmed by monitoring the total energy and radius of gyration (R_g_) of the polymer chains. Data were sampled every 10,000 steps (10 ps) to maintain statistical independence and ensure their reliability for postprocessing.

#### 3.1.2. Single-Particle Model for the Dispersant Layer

A single-particle model was constructed to characterize the adsorbed dispersant layer. Dispersant molecules were anchored to sites on the virtual sphere, which was placed at the center of a 120 Å × 120 Å × 120 Å simulation box. The remaining volume of the simulation box was randomly filled with binder and solvent molecules. The simulation was performed in three stages: (1) energy minimization in 10,000 steps using the conjugate gradient algorithm [46], (2) equilibration in 400 ns with the NVT ensemble at 300 K, and (3) a production run for data collection. The final state at 400 ns was analyzed for structural characterization. The effective thickness was defined as the average height of the adsorbed dispersant layer, calculated as the distance from the surface of the virtual sphere to the median position for the terminal atoms of the dispersant molecules. Crystallinity was calculated as the ratio of crystallized dispersant molecules to total dispersant molecules [34]. A dispersant molecule was defined as crystallized if it satisfied two structural criteria: the end-to-end length of the adsorbed chain reaches at least 90% of its fully extended length, and the terminal atom of the chain has a minimum of three neighboring terminal atoms within a cutoff distance of 5 Å, which corresponds to the first coordination shell cutoff of the radial distribution function.

#### 3.1.3. Two-Particle Model for Potential of Mean Force Analysis

To quantify the repulsive energy barrier between particles, a two-particle model was constructed in a 240 Å × 120 Å × 120 Å simulation box. Two identical virtual spheres, each with an adsorbed dispersant layer, were placed along the *x*-axis. The PMF between the virtual spheres was calculated via the Blue Moon ensemble (constrained MD) method [25,26]. The surface-to-surface distance (R) between the two virtual spheres was defined as the reaction coordinate and varied from 70 Å (non-interacting state) to 4 Å (compressed state) in 3 Å increments. At each distance, the model was equilibrated for 4 ns, which was followed by a production run for 5 ns. The mean constraint force F(s) required to maintain fixed separation was sampled. The PMF profile was obtained by integrating the mean constraint force using the trapezoidal rule. To ensure reproducibility and statistical reliability of the results, the final PMF profile was derived by averaging the data from 12 independent simulation trials. Each trial was initiated with different random velocity distributions to ensure diverse sampling of the simulation box. Error bars in the PMF profiles represented the 95% confidence interval (CI), calculated based on the standard error of the mean across these independent runs:

$$PMF=\langle -\int_{R_{max}}^{R}{{\langle F(s)\rangle }_{t}ds\rangle }_{ens}$$

where 〈*F*(*s*)〉*_t_* denotes the time-averaged constraint force sampled during the production run at a separation distance *s*, and the subscript *ens* indicates the ensemble average obtained from 12 independent simulation trials.

### 3.2. Experimental Methods

#### 3.2.1. Materials

Nickel pastes were formulated from high-purity nickel powder, ethyl cellulose (EC) binder, and dihydroterpinyl acetate (DHTA). Three fatty acids—stearic, lauric, and oleic acid—were employed as dispersants, each added at 0.15 wt% relative to nickel powder. This concentration was selected to be lower than the adsorption capacities of fatty acids on nickel and nickel oxide surfaces reported in previous studies [17,37,38,39], thereby aiming to establish a controlled partial-coverage condition while minimizing the presence of unadsorbed dispersant in the bulk solvent. Therefore, both simulations and experiments were performed under this condition to clearly investigate the effect of adsorbed dispersant structure on dispersibility. Due to corporate proprietary policies, the detailed compositions and suppliers of these components are not disclosed here. To ensure highly dispersed nickel pastes, pre-mixing and three-roll milling processes were employed.

#### 3.2.2. Cryogenic Focused Ion Beam-Scanning Electron Microscopy

The internal morphology and dispersion state were analyzed by Cryo FIB-SEM (Carl Zeiss Crossbeam 550) at the Reliability Assessment Center for Chemical Materials in the Korea Research Institute of Chemical Technology (KRICT). Samples were rapidly vitrified at −160 °C to preserve the native spatial arrangement of the paste [35,36]. Cross-sections with a width of ~30 µm and depth of 7–10 µm were prepared via in situ focused ion beam milling. Over 1000 nickel powder particles were captured per sample to ensure statistical significance. The agglomeration ratio was quantified through image processing of the resulting micrographs [47]. Nickel powder particles were defined as agglomerated if in direct contact or within a proximity threshold, which was set to the median particle diameter (~137 nm). The agglomeration ratio is calculated as the ratio of the number of agglomerated particles divided by the total number of particles within the cross-sectional area. Based on more than 1,000 nickel powder particles, the binomial proportion confidence interval was calculated as the statistical margin of error [48,49,50]. The standard error (*SE*) was computed as $SE=\sqrt{\frac{p(1-p)}{n}}$, where *p* is the aggregation ratio, and *n* is the number of nickel powder particles. To assess a 95% confidence level, we computed the interval as 1.96 × *SE*, displayed as the error bars in Figure 4.

## 4. Conclusions

Our results showed that a thick and highly crystalline adsorbed dispersant layer leads to effective steric stabilization of nickel pastes, which is an insight that should help accelerate dispersant design for nickel pastes for fabricating inner electrodes in MLCCs.

While this study successfully decoupled the effects of chain length and double bonds of a single dispersant within the DHTA-based nickel pastes, broader application may require further investigations. For instance, in more polar solvents where the solubility of stearic acid (C18) decreases, shorter dispersants like palmitic acid (C16) may remain soluble and thus potentially provide better dispersibility. Furthermore, to achieve an even thicker and more highly crystalline layer, future studies should explore the effect of mixed dispersants with various anchoring groups (e.g., acidic/basic) to effectively cover the nickel/nickel oxide surface and fill the interstitial spaces within the dispersant layer.

Although this approach provides molecular-level insights, the difference in particle size and surface curvature between the simulation model and the experimental powder limits direct quantitative correlation with experimental data. In addition, the current atomistic model cannot predict the behavior and rheological properties of powder mixtures within pastes. As a next step, we plan to scale up our model by using coarse-grained MD to analyze the agglomeration and rheology of powder mixtures within pastes to enable quantitative comparison with experimental measurements.

## Data Availability

The data presented in this study are available from the corresponding authors upon reasonable request.

## Abbreviations

The following abbreviations are used in this manuscript:

MLCC	Multilayer ceramic capacitor
MD	Molecular dynamics
PMF	Potential of mean force
Cryo FIB-SEM	Cryo-focused ion beam-scanning electron microscopy
